# Reduced neural responses to reward reflect anhedonia and inattention: an ERP study

**DOI:** 10.1038/s41598-022-21591-9

**Published:** 2022-10-19

**Authors:** Zhengjie Liu, Mengyun Wang, Xiaojuan Zhou, Shubao Qin, Ziyang Zeng, Zhongming Zhang

**Affiliations:** 1 Faculty of Psychology, Southwest University, Chongqing, 400715 China; 2 Department of Biological and Medical Psychology, University of Bergen, Bergen, 5000 Norway

**Keywords:** Diseases, Psychiatric disorders, Biomarkers, Diagnostic markers

## Abstract

An inhibited neural response to reward is typical of clinical depression and can predict an individual's overall depressive symptoms. However, the mechanism underlying this are unclear. Previous studies have found that anhedonia and inattention may mediate the relationship between reward sensitivity and depressive symptoms. Therefore, this study aimed to verify the relationship between reward sensitivity and overall depressive symptoms in a depressive tendency sample as well as to explore the mechanism underlying the ability of neural responses to reward to predict overall depressive symptoms via a mediation model. Sixty-four participants (33 with depressive tendencies and 31 without; dichotomized by BDI-II) finished simple gambling tasks while their event-related potential components (ERPs) were recorded and compared. Linear regression was conducted to verify the predictive effect of ERPs on overall depressive symptoms. A multiple mediator model was used, with anhedonia and distractibility as mediators reward sensitivity and overall depressive symptoms. The amplitude of reward positivity (ΔRewP) was greater in healthy controls compared to those with depressive tendencies (*p* = 0.006). Both the gain-locked ERP component (*b* = − 1.183, *p* = 0.007) and the ΔRewP (*b* = − 0.991, *p* = 0.024) could significantly negatively predict overall depressive symptoms even after controlling for all anxiety symptoms. The indirect effects of anhedonia and distractibility were significant (both confidence intervals did not contain 0) while the direct effect of reward sensitivity on depressive symptom was not significant (lower confidence interval = − 0.320, upper confidence interval = 0.065). Individuals with depressive tendencies display impaired neural responses to reward compared to healthy controls and reduced individual neural responses to reward may reflect the different biotypes of depression such as anhedonia and inattention.

## Introduction

Depression is one of the most common psychiatric disorders, causing severe suffering and disability worldwide^1^. Previous studies have found that many depressive symptoms are driven by deficits in the reward circuit, also known as the positive affect circuit^2,3^. In fact, numerous clinical studies have observed inhibited neural responses to rewarding stimuli^4–6^. Notably, the reward-related positivity^7^ (ΔRewP) is an event-related potential (ERP) elicited by simple gambling tasks^8^, that is considered to reflect one’s reward sensitivity, and positively correlates with activation of the ventral striatum and dorsal anterior cingulate^9^. The ΔRewP is always indexed by the difference in feedback-locked ERPs between rewarding and non-rewarding stimuli (gain-locked ERP minus loss-locked ERP). Importantly, the ΔRewP amplitude has been found to be an effective predictor of individual depressive symptoms in adolescents and preschoolers^10–12^. Additionally, a recent meta-analysis of 21 studies also found weak but significant statistical power (around 27%) of the ΔRewP as a biomarker for depression^13^. Despite the explicit evidence that ΔRewP amplitude can predict depressive symptoms, the specific mechanism(s) mediating the relationship between reward sensitivity and depressive symptoms remains unclear. Uncovering these mechanisms is crucial to the field as they could lead to the development of more targeted interventions.

Anhedonia, one of the main characteristics of various psychiatric disorders^14^, is often described as the loss of pleasure or lack of reactivity to pleasurable stimuli^15^. As a kind of maladaptive clinical symptom^16^, anhedonia is considered to reflect deficits in hedonic capacity closely linked to the constructs of reward valuation, decision-making, anticipation, and motivation^17^, which can be either a personality trait or a event-induced transient state. Lack of pleasant experiences is a typical cause of depression and frequently indicates depressive symptom aggravation or a worsening prognosis^18,19^. Importantly, neuroimaging studies suggest that a dysfunctional reward circuit may result in anhedonia^20,21^. Furthermore, numerous electroencephalogram (EEG) studies have found that decreased neural responses to reward, such as ΔRewP and Feedback Negativity (FN), are related to anhedonia^22,23^. Liu et al.^5^ found that the amplitude of FN to gain feedback in participants with depression was related to anhedonia severity. Reward feedback related ERP components were negatively associated with anticipatory anhedonia^24,25^. A clinical study showed that the ΔRewP of depression patients in the psychological intervention group was significantly enhanced while the symptoms of anhedonia were reduced^10^. It suggested the causal relationship between the ΔRewP and anhedonia, because the amplitude change of the ΔRewP (instead of the symptoms of anhedonia) directly reflects the effect of psychological intervention on the brain reward system. In addition, similar to the prediction of the ΔRewP for depressive symptoms, it is reasonable to assume that anhedonia, as a phenotype of depression, can be predicted by the ΔRewP. Therefore, anhedonia may be a mediator between reward sensitivity and overall depressive symptoms.

In addition to abnormal reward circuits, dysfunctional attention circuits (especially the frontoparietal area) have also been reported in individuals with depression^3^. Inattention or distractibility (inability to sustain attention) is another prevailing maladaptive clinical symptom that is extremely relevant to major depressive disorder (MDD). In fact, previous studies have reported attention deficits in patients with depression^26^ and that these depression and distraction symptoms (such as distractibility) are connected^27,28^. Evidence from functional magnetic resonance imaging (fMRI) studies suggests that the selective attention impairment observed in MDD patients is associated with the intrinsic hypo-connectivity of their frontoparietal attention network^29^. Among EEG studies, a longer mismatch negativity (MMN) latency and smaller P300 amplitude have been found to be related to distractibility^30–32^. Furthermore, the ΔRewP was found to predict adolescents’ initial attention deficit and hyperactivity disorder (ADHD) symptoms in a cross-sectional ERP study^31^. An ERP study showed that the ΔRewP can predict one’s depression symptoms by the mediating role of behavioral inhibition^23^. According to the disinhibited cognitive profile, high reward-sensitive individuals would show a reduced attention to unexpected stimuli^33^. Reduced reward sensitivity may lead to disinhibited behavior such as distractibility^34^. Similar to anhedonia, distractibility is also a kind of depressive phenotype, which can predict the increase of depressive symptoms. Taken together, these findings suggest that distractibility may mediate the path from the reward sensitivity to overall depressive symptoms in a distinct brain circuit from that of anhedonia.

Given that psychiatric medications may affect ERP results^35,36^, the primary goal of the current study was to verify the relationship between reward sensitivity and overall depressive symptoms in a cohort of medication-naive patients with depressive tendencies (no subjects had taken psychiatric medications in the past). We compared the ERPs induced by simple gambling tasks between individuals with depressive tendencies and those without (healthy controls). Understanding the mechanisms underlying how reward sensitivity predicts an individual’s depressive symptoms is also crucial to the prevention of depression. Therefore, to explore the mechanisms mediating this relationship, we investigated the role of anhedonia and distractibility. These two factors were incorporated as mediators in a path analysis to elucidate how reward ERPs can predict overall depressive symptoms.

## Methods

### Participants

Participants were recruited on campus via an online questionnaire or advertisement. A total of 318 students completed the questionnaire and 64 of them were invited to participate in the subsequent EEG experiment. Students with the Beck Depression Inventory Second Edition (BDI-II) scores > 13^37^ (considered to reflect depressive tendencies) were allocated to the group with depressive tendencies (DT group, *n* = 33; mild 12, moderate 6, severe 15) and the rest (scores ≤ 13) were labelled as healthy controls (HC group, *n* = 31). Exclusion criteria for both the DT and HC groups were: (1) history of head injury with possible neurological sequelae; (2) substance abuse or dependence in the past 6 months; and (3) history of psychiatric medications. There was no significant difference between the two groups with respect to age or sex.

Participants were told about the content of the experiment and written informed consent was obtained prior to the experiment. All methods of the study were in accordance with the Helsinki guidelines. The experimental protocol was approved by Institutional Review Board of Faculty of Psychology, Southwest University.

### Measures

All participants rated their overall depressive symptoms using the BDI-II, which consists of 21 items^37^. The BDI-II total score ranges from 0 to 63, with a higher score indicating a higher level of depressive tendencies. In the current sample, the internal BDI-II consistency was excellent (Cronbach’s α = 0.945).

Considering the high correlation between depression and anxiety, participants also rated their overall anxiety symptoms with the Self-Rating Anxiety Scale^38^ (SAS). The SAS is a 4-point Likert scale with 20 items, where a higher total score indicates a higher level of anxiety. In the current sample, the internal consistency of the SAS was good (Cronbach’s α = 0.871).

To measure the severity of individual anhedonia and inattention, participants completed the anhedonia and distractibility subscales of the DSM-5 personality inventory^16^ (PID5). These two subscales contain 8 and 9 items, respectively, and both had good internal consistencies (PID5-anhedonia; Cronbach’s α = 0.852; PID5-distractibility; Cronbach’s α = 0.874). A high score on these two subscales indicates high levels of anhedonia and distractibility.

### Procedure

**Figure 1:**
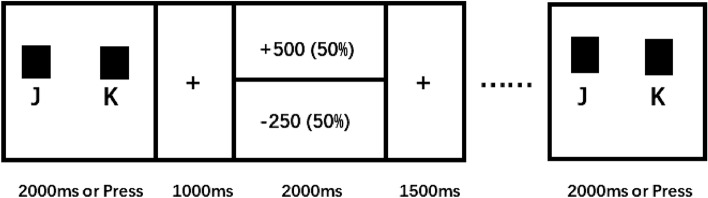
Diagram outlining the experimental design (arrows were displayed in the actual experiment feedback instead of numbers).

Before the EEG task, participants completed self-report questionnaires. The EEG task was a simple gambling task^8^ consisting of two blocks of 45 trials each. The timing diagram is shown in Fig. 1. For each trial, participants were shown two identical boxes and had to select one by pressing the J or K key on a keyboard. A fixation cross was presented for 1000 ms after pressing, followed by a feedback stimulus (gain or loss) presented for 2000 ms. Then, another fixation cross was displayed for 1500 ms. In the feedback interface, arrows pointing upward represented a gain of 500 game points while arrows pointing downward represented a loss of 250 game points. Feedback stimuli were presented pseudo-randomly in each block to assure equal representation of gains and losses. Participants were informed that the game points for each trial were cumulative and that they should try to win as many points as possible to improve their remuneration^4^.

### EEG recordings and analysis

The EEG was recorded using a 64-channel amplifier with a sampling frequency of 500 Hz (Brain Products, Gilching, Germany). The impedance of all electrodes was < 5 KΩ. EEG data were analysed with the EEGLAB (version 8.10) MATLAB (version R2020b) toolbox^39^. Electrode FCz served as reference off-line re-referenced to the average of the left and right mastoids. The recorded EEG data were filtered from 0.01 to 30 Hz. Feedback-locked epochs with a duration of 1000 ms starting 200 ms before feedback presentation were extracted. Eyeblink and ocular-movement artefacts were removed using an automated approach based on independent component analysis^40^. Epochs containing a voltage > 50 μV between consecutive sample points were automatically rejected. Each participants’ rejection rate was < 25% (Each participants remained at least 35 trials for each feedback type). Additional artefacts were removed based on visual inspection. Baseline-correction was applied using a 200 ms pre-stimulus interval. After referring to previous studies and visual inspection of waveforms in the current study, Feedback-locked ERPs were calculated as the mean amplitudes from 250 to 350 ms^23^ after feedback presentation at FCz^41^.

### Statistical analysis

Analyses were conducted with IBM SPSS Statistics, version 22.0 (IBM, Armonk, NY) and Mplus, version 8.3 (Muthe´n LK & Muthen BO, Los Angeles, CA). The significance level was set at *α* = 0.05. The demographic and self-report data were analysed with *t* tests, apart from sex, which was analysed with a *χ*^2^ test. A two-way repeated-measures analysis of variance (rANOVA) with group (DT group, HC group) as the between-subject factor and feedback type (gain, loss) as the within-subject factor was used for ERPs on gain and loss trials and an independent-sample t test was performed for the ΔRewP (calculated as gain minus loss). Feedback-locked ERPs of gain and loss were input into the linear regression model as predictors of overall depressive symptoms, either separately or simultaneously. Hierarchical multiple regression was used to investigate weather ERPs can predict depressive symptoms after controlling for anxiety symptoms. Bivariate correlations were assessed using Pearson’s r correlations among self-report data and ERPs. Multiple comparison correction based on Benjamini–Hochberg false discovery rate (BHFDR) was conducted to adjust the significance level of a single test. Harman’s single-factor test was conducted to verified whether there were common method bias. If correlations among variables were significant, mediation analyses were conducted using the Mplus, with anhedonia and distractibility as mediators. All mediation models used a bias corrected bootstrapping approach (bcbootstrap) with 5000 bootstrapped samples, and significance was determined using a 95% confidence interval.

## Results

### Demographics and self-reported data

There were no significant group differences with respect to age or sex (all *p* > 0.216). Self-reported depressive and anxiety symptoms were significantly higher in the DT group compared to the HC group (all *p* ≤ 0.001). Anhedonia and distractibility PID5 subscale scores were also significantly higher in the DT group compared to those in the HC group (all *p* < 0.001, see Table 1).

**Table 1:** Demographics, self-reported data and the ΔRewP for the participants with/without depressive tendencies.

	DT group (*n* = 33)	HC group (*n* = 31)	*t* (*df* = 62)	*p*	*g*
Age, years	20.76 (1.75)	20.75 (1.75)	0.032	0.975	0.01
Female, number (%)	20 (60.6%)	14 (45.2%)	1.531 (χ^2^)	0.216	0.12 (*Φ*)
BDI	25.24 (7.90)	2.45 (1.79)	15.672	< 0.001	3.92
SAS	46.09 (10.81)	38.06 (7.36)	3.448	0.001	0.86
PID5-anhedonia	9.91 (4.04)	4.81 (2.68)	5.915	< 0.001	1.48
PID5-distractibility	14.39 (4.71)	8.29 (2.48)	6.425	< 0.001	1.61
ERP_gain, μV	6.61 (6.39)	10.41 (5.07)	− 2.628	0.011	0.66
ERP_loss, μV	6.01 (6.12)	7.58 (5.31)	− 1.093	0.279	0.27
ΔRewP, μV	0.6 (3.34)	2.83 (2.91)	− 2.848	0.006	0.71

*DT group* group of participants with depressive tendency, *HC group* group of participants without depressive tendency, *BDI* Beck Depression Inventory-II, *SAS* Self-Rating Anxiety Scale, *PID5-Anhedonia* the anhedonia subscale of the personality inventory for DSM-5, *PID5-Distractibility* the distractibility subscale of the personality inventory for DSM-5, *ΔRewP* difference of mean amplitudes 250–350 ms after gain and loss feedback at FCz. Values are mean (SD); *g*, effect size.

### ERPs

**Figure 2:**
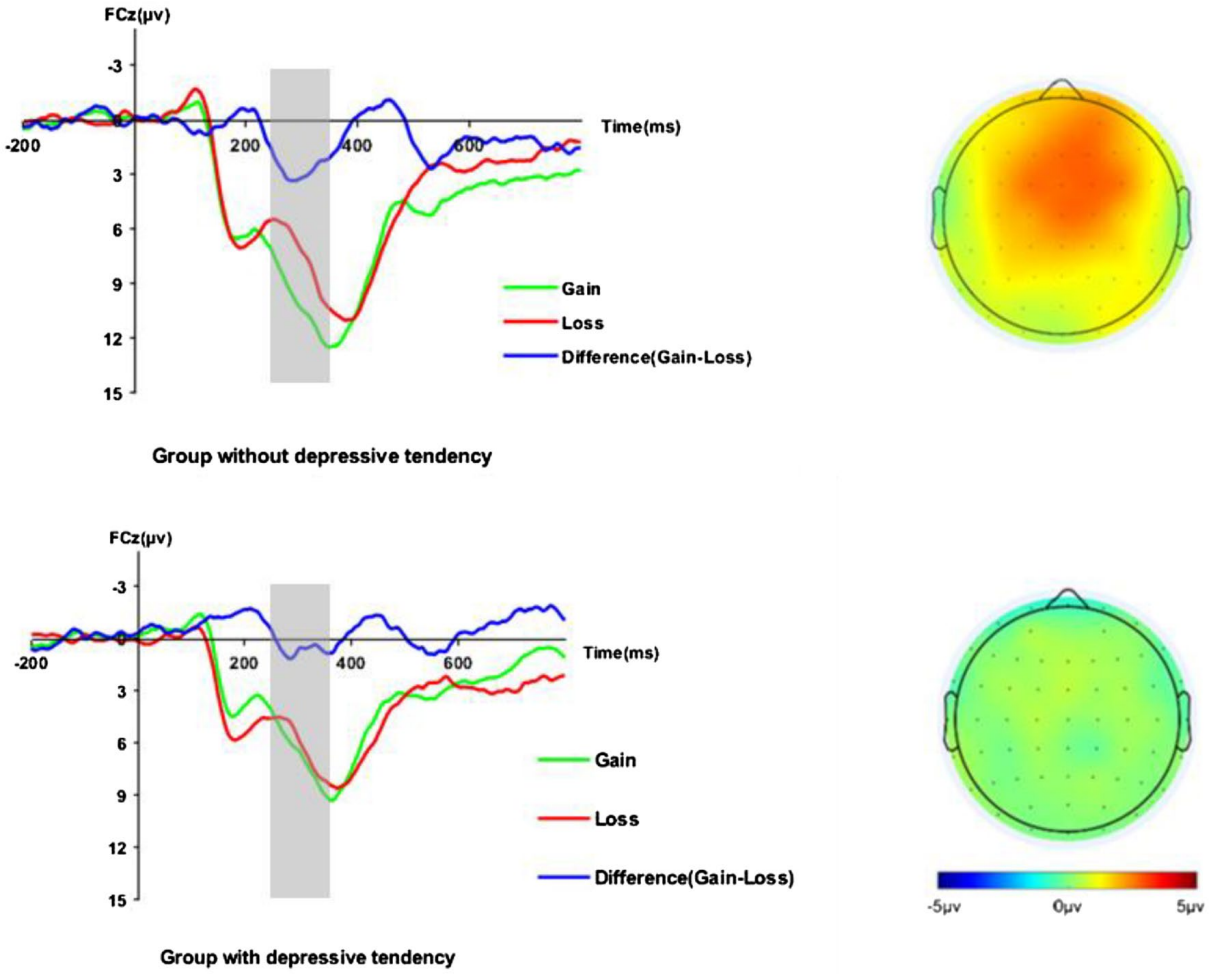
Left: Grand average waveforms for gain, loss and difference (ΔRewP). Right: Headmaps displaying the scalp distribution for the gain–loss difference 250–350 ms post-feedback among the participants with depressive tendencies and those without, respectively.

**Figure 3:**
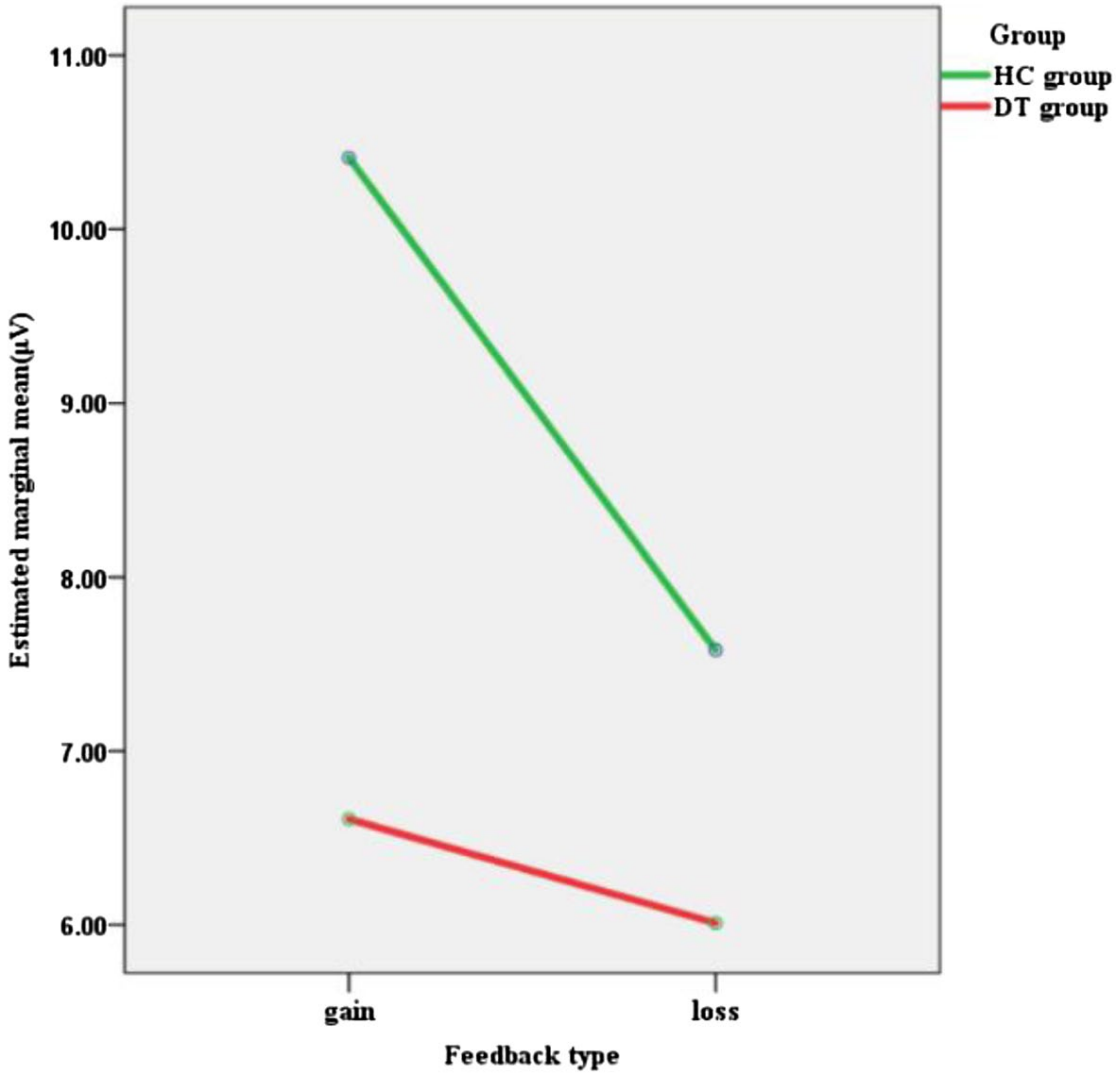
Feedback interaction by group.

**Figure 4:**
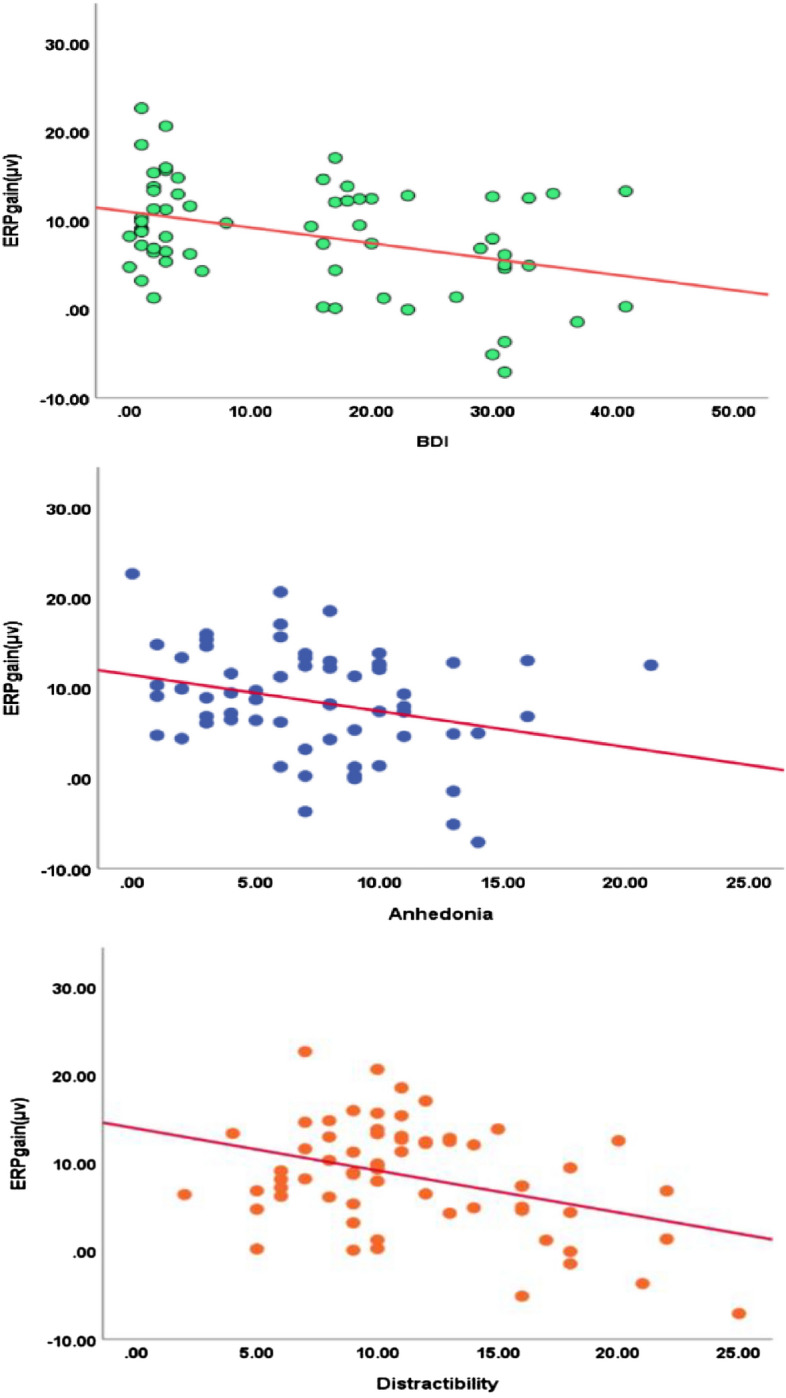
The scatter plots between ERPgain and self-reported data.

Significantly lower gain-locked ERPs were observed in the DT group compared to the HC group (*p* = 0.011, see Table 1), while the loss-locked ERPs were not significantly different between the groups (*p* = 0.279), and the Guttman split-half reliability of the gain-locked and loss-locked ERPs was 0.915. The ΔRewP amplitude was significantly lower in the DT group compared to the HC group (*p* = 0.006). The grand average ERP waveforms for the feedback are shown in Fig. 2. With regards to the rANOVA results, there was a significant main effect of feedback (*F* (1,62) = 19.108, *p* < 0.001, partial *η*^2^ = 0.236). Gain trials (*M* = 8.45, *SD* = 6.05) elicited larger feedback-locked ERPs than loss trials (*M* = 6.77, *SD* = 5.75). The interaction of feedback by group was also significant (*F* (1,62) = 8.111, *p* = 0.006, partial *η*^2^ = 0.116). Specifically, significantly larger ERPs were elicited by gain feedback than loss feedback in the HC group (*p* = 0.004), but not in the DT group (*p* = 0.594). The interaction of feedback by group is shown in Fig. 3. Table 2 displays the bivariate correlations among the self-reported data and ERPs (the scatter plots between ERPgain and self-reported data were presented in Fig. 4).

**Table 2:** Bivariate correlations between the self-reported data and ERPs.

	BDI	SAS	PID5-Anhedonia	PID5-Distractibility	ERP_gain	ERP_loss
BDI	–	0.512**	0.67**	0.628**	− 0.375**	− 0.174
SAS		–	0.524**	0.540**	− 0.179	− 0.017
PID5-Anhedonia			–	0.611**	− 0.282*	− 0.176
PID5-Distractibility				–	− 0.384**	− 0.261*
ERP_gain					–	0.844**
ERP_loss						–

*BDI* Beck Depression Inventory, *SAS* Self-Rating Anxiety Scale, *PID5* DSM-5 personality inventory, *ERP_gain* gain-feedback-locked ERP component, *ERP_loss* loss-feedback-locked ERP component, *ΔRewP* reward positivity. *p < 0.05; **p < 0.01 (correction for multiple comparisons based on BHFDR).

Linear regression models using ERPs as predictors of overall depressive symptoms indicated significant results (see Table 3). In the univariate regression models, both the gain-feedback-locked ERP component and the ΔRewP could negatively predict overall depressive symptoms (all *p* = 0.002), while the loss-feedback-locked ERP component couldn’t (*p* = 0.170). When both gain-locked and loss-locked ERP components were simultaneously input into the multivariable regression model as predictors (the ΔRewP was excluded for its high multicollinearity with the gain-locked ERP), the model became significant (*p* = 0.01). Both gain (*b* = − 1.691, *p* < 0.001) and loss (*b* = 1.114, *p* = 0.022) could significantly predict overall depressive symptoms. The hierarchical multiple regression models showed that the negative prediction of overall depressive symptoms from the gain-locked ERP component (*b* = − 1.183, *p* = 0.007) and the ΔRewP (*b* = − 0.991, *p* = 0.024) were still significant even after controlling for the influence of anxiety symptoms.

**Table 3:** Results from the linear regression analyses predicting overall depressive symptoms from ERPs.

Predictor	Prediction of overall depressive symptom (BDI-II)					
	*R*^2^	*F*	*b*	*SE*	*p*	95% CI
**Univariate**						
ERP_gain	0.141	10.172**	− 0.797	0.250	0.002	[− 1.296, − 0.297]
ERP_loss	0.030	1.928	− 0.388	0.279	0.170	[− 0.946, − 0.170]
ΔRewP	0.148	10.785**	− 1.495	0.455	0.002	[− 2.405, − 0.585]
Multivariable1	0.212	8.227*				
ERP_gain			− 1.691	0.45	< 0.001	[− 2.591, − 0.791]
ERP_loss			1.114	0.47	0.022	[0.167, 2.06]
Multivariable2	0.370	11.749**				
ERP_gain			− 1.183	0.426	0.007	[− 2.036, − 0.330]
ERP_loss			0.679	0.441	0.129	[− 0.204, 1.562]
SAS			0.532	0.137	< 0.001	[0.257, 0.806]
Multivariable3	0.321	14.435**				
ΔRewP			− 0.991	0.429	0.024	[− 1.849, − 0.133]
SAS			0.555	0.141	< 0.001	[0.274, 0.837]

*R*^*2*^ effect size of the model, *F* statistical value of *F* test, *b* regression coefficient, *SE* standard error, *CI* confidence interval, *ΔRewP* reward positivity, *BDI-II* Beck Depression Inventory Second Edition, *SAS* Self-Rating Anxiety Scale, *ERP_gain* gain-feedback-locked ERP component, *ERP_loss* loss-feedback-locked ERP component. *p < 0.05; **p < 0.01.

### Mediation models

**Figure 5:**
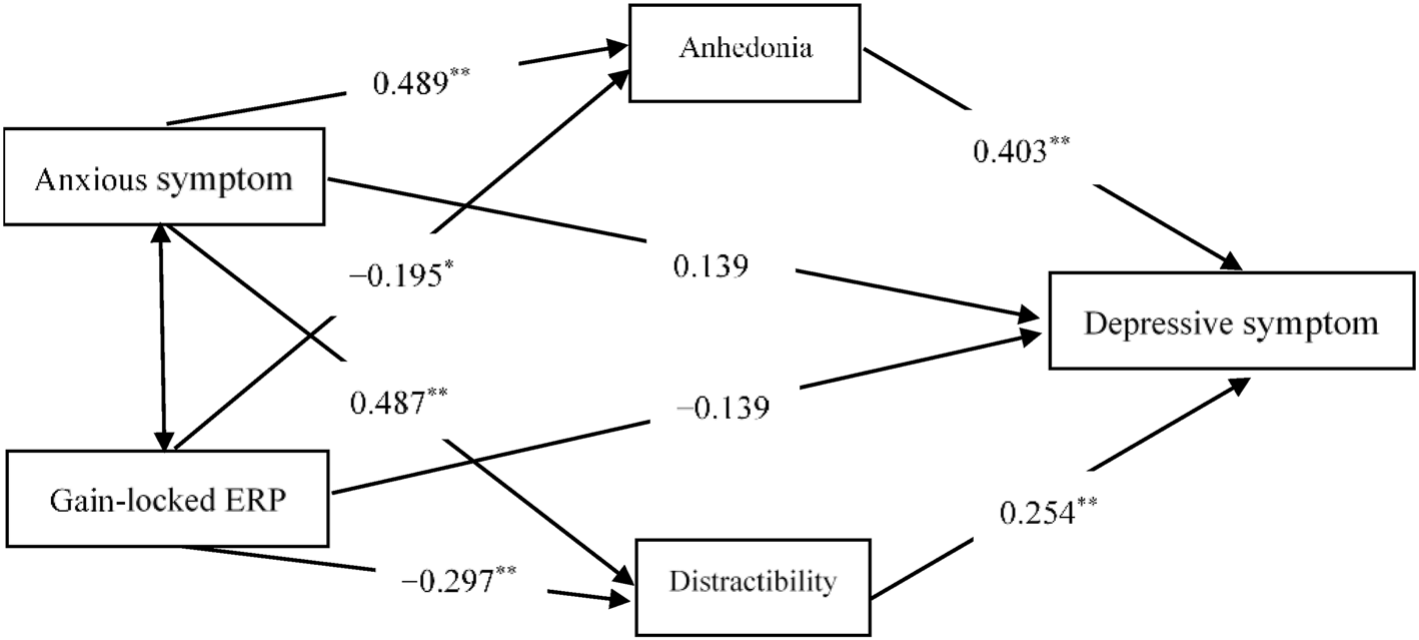
Single-step multiple mediator model of gain-locked ERP component and overall depressive symptoms (*p < 0.05, **p < 0.01).

Harman’s single-factor test showed that the cumulative % of variance was 38.65%, which is lower than 40%. Therefore, there is no common method bias in the current study. Due to the significant correlations observed among the BDI, SAS, PID5-Anhedonia, PID5-Distractibility, and gain-locked ERP component, we first verified the simple mediating effects of anhedonia and distractibility between reward sensitivity and overall depressive symptoms, respectively. Both simple mediator models were significant (see Table 4), so we utilized a multiple mediator model. As the causal relationship between anhedonia and distractibility is not yet clear, we used a single-step multiple mediator model^42^. Given the significant correlations among SAS, BDI, PID5-Anhedonia, and PID5-Distractibility, anxiety symptoms were entered in the single-step multiple mediator model as a covariate. The results of this model are shown in Table 5. The total indirect effect of this model was significant even after controlling for anxiety symptoms (confidence interval did not contain 0). The indirect effect of distractibility was still significant while anhedonia was not (see Table 5). However, the direct effect of the gain-locked ERP component on overall depressive symptoms was not significant (lower confidence interval = − 0.320, upper confidence interval = 0.065). In other words, there is a complete mediation effect in the single-step multiple mediator model. Figure 5 shows the single-step multiple mediator model of gain-locked ERP component and overall depressive symptoms.

**Table 4:** Results from the simple mediator models.

Model	IE estimate	Bootstrap *SE*	Lower CI	Upper CI
Egain-AN-BDI*	− 0.173	0.068	− 0.306	− 0.038
Egain-DI-BDI**	− 0.218	0.074	− 0.372	− 0.078

*CI* confidence interval, *IE* indirect effect, *Egain* gain-feedback-locked ERP component, *AN* Anhedonia, *DI* Distractibility, *BDI* Beck Depression Inventory. *p < 0.05; **p < 0.01.

**Table 5:** Results from the single-step multiple mediator model.

Mediator	IE estimate	Bootstrap *SE*	Lower CI	Upper CI	IE/TE (%)
Anhedonia	− 0.079	0.048	− 0.185	0.003	27
Distractibility*	− 0.075	0.035	− 0.158	− 0.017	25.6
TIE**	− 0.154	0.058	− 0.269	− 0.039	52.6

*CI* confidence interval, *IE* indirect effect, *TE* total effect, *TIE* total indirect effect. *p < 0.05; **p < 0.01.

## Discussion

The present study found that individuals with depressive tendencies are characterized by inhibited overall neural responses to reward feedback compared to healthy controls. This finding is consistent with previous studies which focused on clinical MDD patients^5,6,31^. Given the large number of individuals with depressive tendencies, it is meaningful to conduct this research in such a sample.

It has been hypothesized that anxiety precedes depression, implying an increased risk of depression in individuals suffering from anxiety^43,44^. However, previous studies have largely ignored the influence of anxiety on the predictive power of ERP components for depressive symptoms. In the present study, we found that impaired reward responses could positively predict overall depressive symptom severity even after controlling for the influence of anxiety symptoms. That is, despite the high correlation and comorbidity between depression and anxiety^45^, the inhibited neural responses to reward are independently related to depression^46^. Notably, while the loss-locked ERP component amplitude appeared to positively predict an individual’s overall depressive symptoms in the two-factor multivariate regression model, this effect was lost when anxiety was input into the regression model as a covariate. Therefore, the loss-locked ERP component it is not a good predictor of overall depressive symptoms as it has a high sensitivity to anxiety. Therefore, we recommend the gain-locked ERP component or the ΔRewP as better predictors of overall depressive symptom severity. The advantage of the ΔRewP is that the difference wave can prevent components’ interference^47^; however, the disadvantage is its higher sensitivity to anxiety than the gain-locked ERP component (shown as a smaller regression coefficient in the multiple regression model; Table 3). Since the ΔRewP is calculated by subtracting loss from gain, the high sensitivity to anxiety of the loss-locked ERP component inevitably influences the ΔRewP. Despite the weak influence observed in this study for that component, it supports the value of the gain-locked ERP component as a clearer predictor.

Anhedonia and distractibility were identified as mediators of the relationship between reward sensitivity and an individual’s overall depressive symptom severity. The total indirect effect of these two factors was considerably good, accounting for 52.6% of the total effect. Notably, the impaired neural response to gain implies one’s more serious self-report anhedonia and inattention, which are different biotypes of depression^3^. Dysfunctional reward and attention circuits are related to anhedonia and inattention, respectively. Consistent with the reduced amplitude of neural responses to gain, striatal hypoactivation has been observed in people with depression^48^. Importantly, such deficits in the reward circuit present as anhedonia and finally aggravate individual overall depressive symptoms. Further, hypoconnectivity within the frontoparietal attention circuit has been reported in MDD patients^49^ suggesting the presence of inattention in depression. Interestingly, we also found a negative correlation between the neural responses to gain and distractibility, a relationship poorly investigated in previous studies. As a result, the weakened neural responses to gain indicate deficits in attention circuits (presented as distractibility) that aggravate the individual’s overall depressive symptoms. Finally, the complete mediation effect of anhedonia and distractibility imply that inhibited neural responses to reward reflects depression biotypes such as anhedonia and inattention. Even though anhedonia and distractibility are linked with a single psychophysiological measure indexing in the current study, the ERP components are actually the superposition result of various EEG activities. However, none previous studies have found an association between attention circuitry and the RewP. So, the conclusions of this study still need to be verified by a large number of repeated studies. In terms of the results of this study, when individuals show impaired neural responses to reward, targeted interventions, such as enhancing the sensitivity of pleasure and concentration training, may be useful for reducing the risk of depression and relieving overall depressive symptoms. Of course, this conclusion needs to be verified in future intervention studies.

This study is has some limitations. First, as all participants were recruited on campus, the representativeness of this sample is limited, thus future studies with larger sample sizes from various regions are required. Second, this was a cross-sectional study which only measured participants’ baseline overall depressive symptoms. Although it is difficult to determine the causal relationship between variables in the cross-sectional research, we described our hypothetical model in the introduction and preliminarily verified it through the current study. A longitudinal design should be considered in future studies to replicate the results in the current study. Finally, both anhedonia and distractibility were quantified using self-report scales. Therefore, future studies including behavioural indicators of anhedonia and distractibility are warranted^50^.

## Conclusion

First, individuals with depressive tendencies have impaired neural responses to reward compared to healthy controls. Second, the gain-locked ERP component is a more effective predictor of individual overall depressive symptoms than the loss-locked ERP component and the ΔRewP. Third, reduced individual neural responses to reward may reflect different depression biotypes such as anhedonia and inattention. Taken together, our findings suggest that when individuals show impaired neural responses to reward, targeted interventions, such as enhancing the sensitivity of pleasure and concentration training, may be used to reduce the risk of depression as well as relieve overall depressive symptoms.

## Supplementary Information

Supplementary Information.

## Data Availability

All data generated or analysed during this study are included in this published article (and its supplementary information files).
